# Comparison of Safety Margin Generation Concepts in Image Guided Radiotherapy to Account for Daily Head and Neck Pose Variations

**DOI:** 10.1371/journal.pone.0168916

**Published:** 2016-12-29

**Authors:** Markus Stoll, Eva Maria Stoiber, Sarah Grimm, Jürgen Debus, Rolf Bendl, Kristina Giske

**Affiliations:** 1 Department of Medical Physics in Radiation Oncology, German Cancer Research Center (DKFZ), Heidelberg, Germany; 2 Heidelberg Institute for Radiation Oncology (HIRO), National Center for Radiation Research in Oncology, Heidelberg, Germany; 3 Faculty of Computer Science, Heilbronn University, Heilbronn, Germany; 4 Department of Radiation Oncology, University Hospital, Heidelberg, Germany; North Shore Long Island Jewish Health System, UNITED STATES

## Abstract

**Purpose:**

Intensity modulated radiation therapy (IMRT) of head and neck tumors allows a precise conformation of the high-dose region to clinical target volumes (CTVs) while respecting dose limits to organs a risk (OARs). Accurate patient setup reduces translational and rotational deviations between therapy planning and therapy delivery days. However, uncertainties in the shape of the CTV and OARs due to e.g. small pose variations in the highly deformable anatomy of the head and neck region can still compromise the dose conformation. Routinely applied safety margins around the CTV cause higher dose deposition in adjacent healthy tissue and should be kept as small as possible.

**Materials and Methods:**

In this work we evaluate and compare three approaches for margin generation 1) a clinically used approach with a constant isotropic 3 mm margin, 2) a previously proposed approach adopting a spatial model of the patient and 3) a newly developed approach adopting a biomechanical model of the patient. All approaches are retrospectively evaluated using a large patient cohort of over 500 fraction control CT images with heterogeneous pose changes. Automatic methods for finding landmark positions in the control CT images are combined with a patient specific biomechanical finite element model to evaluate the CTV deformation.

**Results:**

The applied methods for deformation modeling show that the pose changes cause deformations in the target region with a mean motion magnitude of 1.80 mm. We found that the CTV size can be reduced by both variable margin approaches by 15.6% and 13.3% respectively, while maintaining the CTV coverage. With approach 3 an increase of target coverage was obtained.

**Conclusion:**

Variable margins increase target coverage, reduce risk to OARs and improve healthy tissue sparing at the same time.

## Introduction

**Fig 1:**
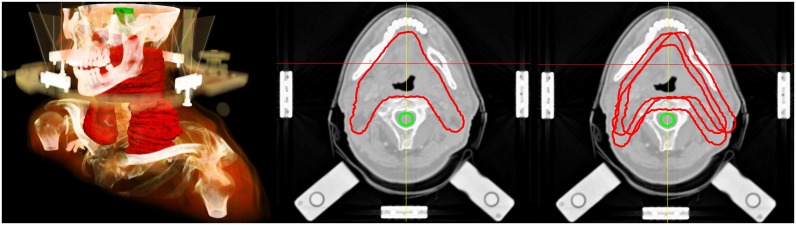
Clinical Target Volume (CTV) of a head and neck cancer patient. Left: Volume rendering of the planning CT image for an exemplary head and neck cancer patient receiving an IMRT treatment under daily image guidance. The clinical target volume (CTV) is displayed in red, brainstem in green. The stereotactic external frame is used as positioning device. Center: A transversal CT slice of the same patient of the mandibular region is displayed. The clinical target volume (CTV, red) and the spinal cord (green) as organ at risk are shown as planning contours. Right: CTV contours of different treatment days are plotted on the same planning CT slice to visualize daily variations.

Radiotherapy has become an effective main or complimentary curative treatment for head and neck tumors [1]. It aims to destroy tumor cells with ionizing radiation. The volume to be irradiated, the clinical target volume (CTV, Fig 1), usually contains the primary tumor and the area surrounding the lymph nodes. The CTV can be in close proximity to healthy organs that are sensitive to high dose deposition like the brainstem or the spinal cord [2]. Those are called organs at risks (OARs) and need to be spared from irradiation. Other healthy surrounding tissue needs to be protected as well, due to significant long-term morbidity potentially caused by irradiation [2].

Intensity modulated radiation therapy (IMRT) in contrast to the conventional 3D planning technique allows a better conformation of the high-dose region to target volumes on the planning CT. This leads to individually tailored target coverage while respecting dose limits to critical OARs [2]. IMRT was proven to increase the quality of life after radiotherapy, reducing xerostomia, as well as other side effects and symptoms [3].

An advanced planning process is required to perform an IMRT. This is realized based on a planning CT image with contoured OARs and the CTVs [4]. Dose constraints like minimum dose to a certain CTV fraction and maximum dose to volume fractions of relevant OARs are prescribed and a radiotherapy plan is automatically optimized in respect to the constraints [5]. Additional details of the sophisticated planning process chain can be found in [4,6].

Head and neck cancer is effectively treated with a fractionated radiotherapy. A fractionated therapy takes advantage of the inferior repair capabilities of tumor cells compared to cells in healthy tissue [7]. Therefore, the treatment is delivered on multiple treatment days and several days after the planning. To ensure that dose is delivered as planned, the patient has to be set up in the same position. Patient setup with laser positioning and stereotactic devices [8] combined with immobilization devices (e.g. thermoplastic mask) allow to reproduce the patient position in the planning pose.

This accurate patient setup reduces changes between therapy planning and delivery but may be affected by uncertainties in position and shape of the CTV and OARs. Reasons for this uncertainties are mainly setup errors of the positioning devices [9], pose variations due to different bone and joint positions, soft tissue shift caused by muscle and organ activites as well as weight loss of the patients [10–13]. While rigid setup uncertainties can be compensated with rigid treatment couch corrections calculated from fraction control CT images in image guided radiation therapy (IGRT) [14], the remaining anatomical deformations of the tumor shape can compromise the dose coverage of the CTV [15,16].

Without adaptive plan procedures [17], these residual uncertainties have to be addressed by the use of internal safety margins around the CTV, creating the internal target volume (ITV) [5]. This concept was initially introduced for fast motions e.g. for lung tumors [18] and adapted for swallow motions in head neck [19]. We propose to use the ITV concept also for daily deformations. In this work we focus on pose variations characterized by different bone configurations.

Additional external changes that derive from technical setup uncertainties are accounted for with an additional setup margin. It is added to the ITV to form the final planning target volume (PTV), which is required in the planning process. In the absence of daily image guidance, a wide spread PTV construction concept was frequently applied (using a homogeneous expansion to the CTV) called van Herk recipe [20,21]. Typical values ranged between 8 and 10 mm [22]. With the introduction of daily image guidance and treatment couch corrections, the total CTV-PTV margin size could be reduced to 3 mm, showing to be sufficient for head and neck treatments [23].

Since the additional safety margins cause higher dose deposition in adjacent healthy tissue inside the PTV, their size should be as large as necessary and simultaneously as small as possible. Concepts to cope with the residual “non-compensable” deformations concern either modified couch corrections reducing the adverse effects [11,16] or a modified heterogeneous margin construction approach [24,25]. Since the amount of deformations in the head and neck anatomy are not distributed uniformly [11,26], the ideal margin generation should consider their local uncertainty distribution [24,25].

In this work we evaluate and compare three approaches for the generation of internal margins to account for daily pose variations 1) a clinically used approach, 2) a previously proposed approach adopting a spatial model of the patient and 3) a newly developed approach adopting a biomechanical model of the patient. All approaches are evaluated using the same large patient cohort of over 500 fraction control CT images with heterogeneous pose changes to enable a comparison of the different effects which could be achieved in the clinical application.

## Materials and Methods

### 2.1 Patient data and target volumes

More than 500 CT images of 19 head and neck cancer patients undergoing IMRT treatment (Artiste, Siemens OCS, Erlangen, Germany) were included in this retrospective study. The patients were treated postoperatively for oropharyngeal cancer or received definitive radiation therapy for locally advanced hypopharyngeal cancer. To reduce pose variation all patients were immobilized with a customized scotch-cast head mask and a vacuum pillow [10]. A stereotactic head frame was used for daily positioning and image guidance was applied with an in-room, on-rails, single slice spiral CT-scanner (Primatom, Siemens, Erlangen). The acquired kilo-voltage fraction control CT images have a resolution of (0.98 x 0.98 x 3 or 2 mm) and near diagnostic quality. Written informed consent to include their anonymized data in retrospective studies was obtained from all patients.

A CTV and a boost volume for an integrated boost concept treatment were delineated in our clinical routine. The CTV contains the pre-surgical gross tumor volume and the supraclavicular and cervical lymph nodes in all patients (Fig 1). In the IMRT planning process the maximum dose to the spinal cord was limited to 45 Gy. The dose prescription to the boosted area containing the primary tumor was 70.4 Gy in 32 fractions (2.2 Gy/fraction) and 57.6 Gy to the CTV. In this study we only use the delineation of spinal cord, brain stem and the CTV for our retrospective evaluation.

### 2.2 Pose variation analysis

**Fig 2:**
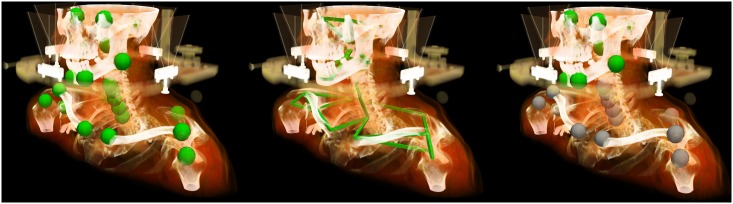
Landmarks were selected in bony structures of the patient. Left: Position of the selected anatomical landmarks located on bony structures; center: geometrical model representing the landmarks connected by rigid-body motion restrictions; right: subset of landmarks which were used for rigid-body Procrustes registration (green) to eliminate setup variations which are typically corrected in IGRT.

We selected 24 anatomical landmarks on bony structures to represent the patient pose (Fig 2, left). All landmarks are located in easily identifiable structures and were manually selected in the planning CT image of each patient. A fully automatic method to find corresponding landmark positions in the control CT images was used. The landmark tracking uses a template matching technique with cross correlation as similarity metric [27]. The normalized cross correlation is used as an absolute measure of conformity, which allows the identification and removal of doubtful correspondences that occur when a landmark is out of the field of view with a correlation coefficient lower than 0.7.

Since landmarks located on the same bones cannot change their distance between each other due to rigid-body motion restriction, those connections are used to build a geometrical model similar to [28]. The probability distributions (mean and covariance matrix) of distances for connected landmarks (Fig 2, center) were calculated. This information is used to calculate the probability of a given landmark displacement in respect to its neighbors displacements and allows identifying landmarks that have been found at wrong positions. Landmarks in the head region with a Mahalanobis distance [29] higher than 15 were removed as well as landmarks near the shoulder with a Mahalanobis distance lower than 60. Based on this outlier analysis, landmarks likely to contradict rigid-body restrictions are removed from the posture variation analysis.

As treatment couch correction can correct rigid translational and rotational positioning variations before the treatment, all control CT images were rigidly preregistered to the corresponding planning CTs before the pose variation analysis. A Procrustes analysis [30] was chosen to simulate this daily IGRT correction procedure using a subset of the landmarks located on the skull of the patient. The calculated transformation was applied on all 24 landmarks using the skull as reference region for deformation analysis (Fig 2, right). The residual displacements at individual landmarks represent the individual deformations.

### 2.3 Deformation modeling with a biomechanical patient model

The residual landmark displacements were used to interpolate a complete displacement vector field (DVF) between the planning CT image and each control CT image. This allows transforming volumes of interest (VOIs) to the fraction control CT images using the DVF. This approach only accounts for changes due to pose variations.

The interpolation is performed with a patient specific biomechanical finite element model. The entire modeling and simulation workflow was formulated with the Medical Simulation Markup Language (MSML) [31]. We provide an example of our workflow with head and neck patient images from the public Cancer Imaging Archive [32,33] in the MSML public repository at https://github.com/CognitionGuidedSurgery/msml/tree/plos-one/examples/HeadNeckExample.

The workflow to construct the biomechanical patient model can be divided into five steps. First, the relevant anatomical structures are segmented in the planning CT image. In radiotherapy often the clinical segmentation from the daily routine can be used. Second, a finite element mesh is generated from the segmented contours. In the third step material properties, boundary conditions and loads are defined and the simulation is performed. In the forth step the resulting DVFs are used for contour propagation. Finally, in the fifth step unknown and uncertain parameter values in the model are calibrated.

Step 1—Segmentation: Only the skin contour available from the clinical routine was used as input segmentation for mesh generation and biomechanical simulation.

Step 2—Mesh generation: CGAL Mesh triangulation refinement [34] was used to generate a 3D volumetric tetrahedral mesh from skin surface segmentation. Applied parameters were facet angle = 20°, facet size = 10 mm, facet distance = 5 mm, cell radius edge ratio = 3 and cell size = 10 mm.

Step 3a –Tissue parameter: We use a homogeneous isotropic linear material [35] for the whole head and neck region. The material properties of tissue are subject to numerous studies. The reported material parameters for certain tissue types disagree. The value of Young’s modulus, that describes the stiffness of material, measured by tensile stretching range from 1 to 19 MPa for liver and kidney and between 2 and 30 MPa for soft tissue in general [36]. Muscles and tendons have a Young’s modulus around 500 MPa and bones can have up to 35,000 MPa. Reported values for Poisson’s ratio (describes the amount of the transversal contraction if a material is stretched) also have a high range with values between 0.29 and 0.49 for different structures in the head [37]. We chose the material parameters in step 5 –Calibration.

Step 3b –Boundary conditions: Force boundary conditions between landmark positions *l*_*i*_ in the FEM model (initially at the landmark position in the planning CT image) and their position in fraction control CT images $l_{i}^{\text{c}}$ are applied. This boundary condition deforms the patient pose from planning CT image to the pose of the fraction control CT image and minimizes the distance

$$d_{i}=l_{i}-l_{i}^{\text{c}}\;\in {\mathbb{R}}^{3}.$$ (1)

During simulation the force

$$f_{i}=k\cdot d_{i}\in {\mathbb{R}}^{3}\;$$ (2)

at each landmark is continuously updated. The spring stiffness k is determined in step 5 –Calibration.

**Fig 3:**
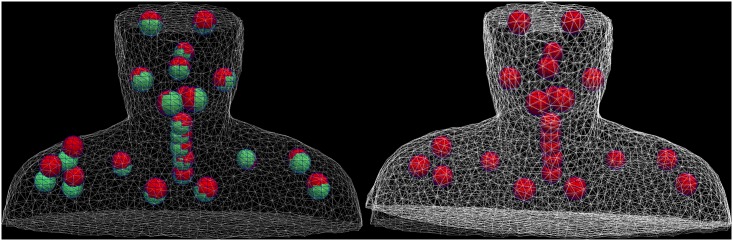
The finite element model of the patient is deformed. Left: the finite element mesh model of the patient’s skin in the planning configuration (grey) and the selected anatomical landmarks (green) as well as their position in the fraction control CT image (red). The patient model is deformed by a force based boundary condition between the landmarks. Right: Landmarks were pulled towards their position in the fraction CT, the mesh is deformed resulting in rising of the right shoulder.

Step 3c –Simulation: The biomechanical simulation is performed in the Simulation Open Framework Architecture (SOFA) [35,38]. A geometrical non-linear method for large deformations with co-rotational finite elements [39] was used. The result of the simulation is a deformed 3D tetrahedral mesh of the patient imitating the pose of the patient in the fraction control CT image (Fig 3).

Step 4 –DVF generation and contour propagation: The deformed and the initial configuration of the tetrahedral mesh can be directly used to propagate meshes from initial to deformed configuration using mapping techniques. We use a simple barycentric mapping approach [31,35]. The same mapping is used to find the DVF for visualization purpose.

Step 5 –Calibration: We initially chose a tissue material with a Young’s modulus of *E* = 10 MPa near the mean of the range reported in the literature [36,37]. Increasing landmark spring stiffness *k* or decreasing the tissue stiffness *E* has similar effects on the resulting DVF. This behavior is comparable to [40], where the choice of different tissue material parameters had only a limited impact on the resulting DVF after the calibration of intrapleural pressure. Thus we calibrate only the landmark spring stiffness *k* and maintain the tissue stiffness *E*. A value for *k* is determined with a single parameter sweep. The residual landmarks pairwise distances are evaluated in this process as a fit criterion. We consider a mean distance of 0.5 mm optimal and determine a corresponding landmark spring stiffness of *k* = 1000 N/mm. With an additional parameter sweep for tissue stiffness *E* we ensure that the simulation results are mesh-independent and stable for *E* = 10 MPa and *k* = 1000 N/mm.

### 2.4 Competing margin generation concepts

Three different approaches were used to create CTV-ITV margins for all 19 patients. A leave-one-out cross-validation was applied where appropriate: the margin for a patient was always created using only fractional information from other patients.

#### 2.4.1 Approach 1: Constant margin

**Fig 4:**
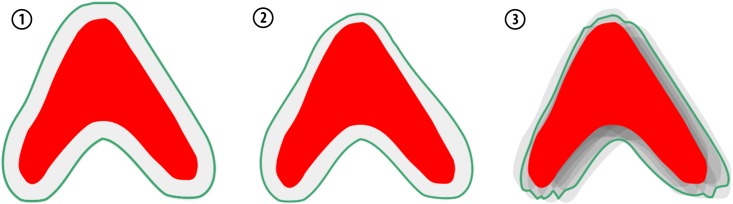
Schematic representation of the competing margin generation concepts to cope with residual deformations after a simulated IGRT correction. Approach 1: The constant margin is applied by increasing the CTV (red) contours by 3 mm resulting in the internal target volume (green). Approach 2: The variable margin is applied locally by increasing the CTV (red) by a value calculated using measured landmark displacements of adjacent landmarks. Approach 3: The variable finite-element-model-based margin is created by statistical sampling of the propagated target volumes based on DVFs generated from landmark displacements by the patient-specific biomechanical model.

A constant CTV-to-PTV margin of 3 mm is often used clinically when IGRT is performed [14,23,41]. We applied this approach for an ITV by extending the CTV homogeneously with a constant safety margin (see Fig 4, left). To achieve this, the 2D contours were converted to a (1x1x1) mm 3D binary voxelized volume and a morphological dilation by a sphere with 3 mm radius was applied [42].

#### 2.4.2 Approach 2: Variable margin based on distance

Yang et al. [24] proposed a method to generate a variable margin including loco-regional variations of setup uncertainties using measured displacements at landmark positions in a collective. The local margin size at a point *x* is calculated as the weighted average

$$r\left(x\right)=\overset{N}{\underset{i=1}{\sum }}w_{i}m_{i}$$ (3)

of margin sizes *m*_*i*_ = 2.5 Σ_i_ + 0.7 *σ*_i_ determined at different landmark positions from previous studies [11] (∑ denotes the systematic setup error and σ denotes the random error [20]). The local margin size was used to create a margin for the CTV. They demonstrated the effectiveness of this approach compared to the constant margin approach.

In order to evaluate this approach, we reimplemented the margin generation algorithm. The distance balancing factor *w*_*i*_ was adapted to the spatial distribution of the landmarks of our patient cohort. The standard deviations (in left-right, dorsal-ventral, and cranial-caudal direction) for each landmark were calculated and a margin based on a confidence interval of 95% was derived.

For the leave-one-out cross-validation the margin for a patient was created using only landmark displacement information from the remaining 18 patients.

#### 2.4.3 Approach 3: Variable margin with biomechanical model

Similar to approach 2, we propose to use landmark displacements measured in a patient collective. Unlike approach 2, where the local landmark displacements are analyzed independently from another, we propose to combine them. For each patient *i* a set of *M* landmarks

$$p_{i}\left(f\right)=(l_{1}\left(f\right)\ldots l_{N}\left(f\right)\in {\mathbb{R}}^{3M}$$ (4)

of fraction f is built and the poses

$$u_{i}\left(f\right)=p_{i}\left(f\right)-{\bar{p}}_{i}$$ (5)

with mean landmark positions ${\bar{p}}_{i}$, are extracted. We combine all 500 poses in a single statistical pose model using a probabilistic principle component analysis (PPCA [43,44]) to extract the first five eigenvectors *b*_1_ … *b*_5_ of all poses. A pose can then be represented as linear combination of the first five eigenvectors

$$u_{i}\left(f\right)\approx \left(\begin{array}{c} b_{1}\\ \vdots \\ b_{5} \end{array}\right)\cdot \left(q_{1}\cdots q_{5}\right)+\bar{u},$$ (6)

with the coefficients *q*_1_ … *q*_5_. This model considers correlations between landmark displacements and can only fit poses of consistent landmark displacements. For example it cannot fit a fictional pose where landmarks on the same bone would increase their distance. Similar studies were performed for patient specific modeling of motion in the pelvic area [45].

From the statistical pose model we randomly sample a large number (N = 400) of possible pose scenarios using a random number generator for the coefficients *q*_1_ … *q*_5_. Each pose scenario is applied on the biomechanical model of the new patient. The landmarks are pulled from their position in the planning CT image to the synthesized fraction position in order to create motion scenarios (N = 400). This is implemented using the same workflow as described in 2.3. Using the DVF, the initial segmentations of the CTV are deformed and converted to a binary voxel image. In an accumulation image all 400 CTV segmentations are summed to extract all voxels hit in more than 5% of all scenarios.

We have seen that noise reduction for the calculation of mean landmark positions ${\bar{p}}_{i}$ in Eq (5) improves the quality of sampling and we introduced a PPCA based noise reduction akin to the pose model in Eq (6) for the mean poses. A mean landmark position model

$${\bar{p}}_{i}\approx \left(\begin{array}{c} c_{1}\\ \vdots \\ c_{5} \end{array}\right)\cdot \left(r_{1}\cdots r_{5}\right)+\bar{\bar{p}}.$$ (7)

for each patient is fitted with *r*_1_ … *r*_5_ and a noise reduced mean landmark position is reconstructed with Eq (7).

For the leave-one-out cross-validation the statistical pose model is created individually for each patient using only landmark displacement information from the remaining 18 patients.

#### 2.4.4 Margin evaluation

**Fig 5:**
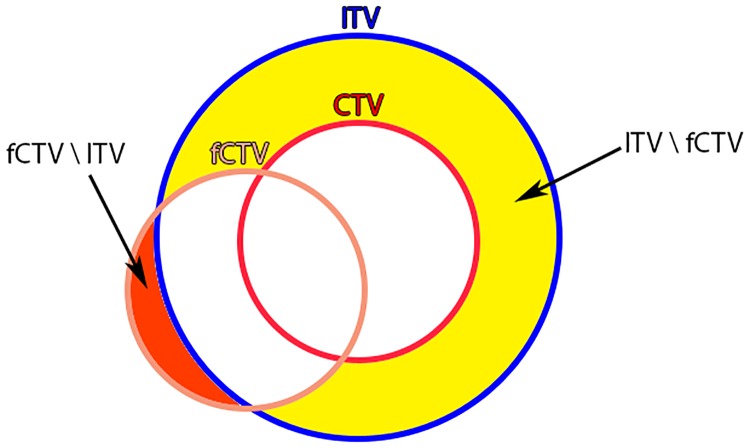
Margin evaluation measurements. The CTV (red) is propagated to the fraction control CT image (fCTV, orange) to calculate CTV volume missed by the treatment ***V*_fCTV \ ITV_** and the volume of healthy tissue being hit $\bar{V_{ITV\;\backslash \;fCTV}}$.

To compare the margin approaches quantitatively, we use four measurements to evaluate the different concepts (Fig 5).

The total size of the generated internal margin volume (*V*_ITV \ CTV_) indicates how much healthy tissue is expected to be irradiated from the planning phase perspective. We measure it by counting all voxels inside the ITV but not inside the CTV in the planning phase.

The mean healthy tissue hit ($\bar{V_{\text{ITV }\backslash \text{ fCTV}}}$) indicates how much healthy tissue was actually irradiated over all fractions. It can be larger than *V*_ITV \ CTV_ in case the CTV moves out of the ITV. We measure it by propagating the CTV to all fractions and counting all voxels inside the ITV that are not inside the propagated fCTV.

The mean missed CTV ($\bar{V_{\;\text{fCTV }\backslash \text{ ITV}}}$) volume indicates how bad the CTV coverage was over all fractions. We measure it by propagating the CTV to all fractions and counting all voxels inside the propagated fCTV that are not inside the ITV.

**Fig 6:**
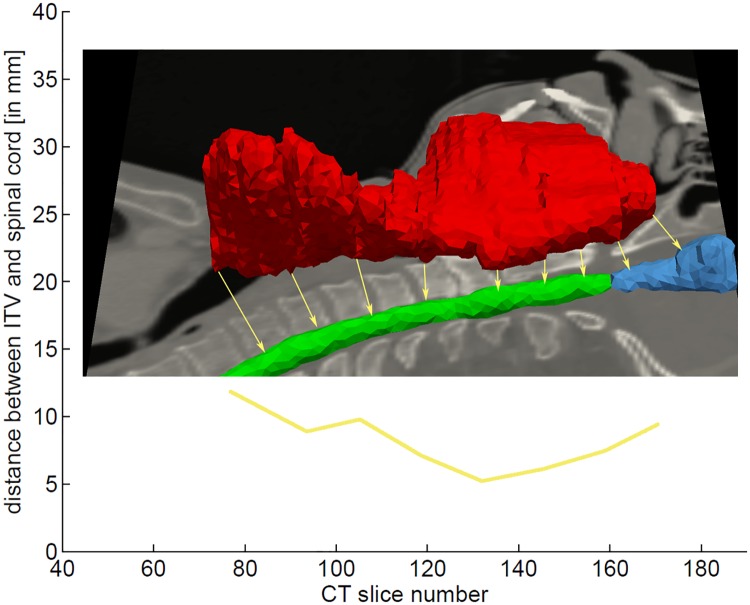
Distance between ITV and OARs per CT slice. The slice wise distance between ITV and spinal cord ***d***_**SC**_**(*****z*****)** is used to estimate the risk to the spinal cord.

The distance between ITV and OARs indicates exposure risk. We measure it exemplary for the spinal cord. It is measured slice wise: The 3D distance to the nearest point in the spinal cord segmentation *d*_SC_(*z*) is measured for each transversal slice of the ITV. *d*_SC_(*z*) is calculated individually for each fraction. Therefore the contours of the spinal cord were propagated to all fractions. Fig 6 shows *d*_SC_(*z*) of a single fraction.

## Results

### 3.1 Pose variation analysis

All 24 landmarks were tracked trough all fraction control CT images of each patient. 6012 out of 36216 landmark positions were removed due to the limited field of view in control CT images or doubtful correspondences detected by outlier detection.

Procrustes analysis matched the skull landmarks with a mean error of less than 1 mm for all control CT images. The mean displacement magnitude of the found translation in left-right, dorsal-ventral and cranial-caudal direction were: (0.73 +- 0.64) mm, (1.08 +- 0.98) mm, and (1.76 +- 1.70) mm, respectively. Mean absolute rotation about left-right, dorsal-ventral, and cranial-caudal axis were: (1.06 +- 0.92°, (0.70 +- 0.65°, and (0.92 +- 1.00°, respectively.

**Fig 7:**
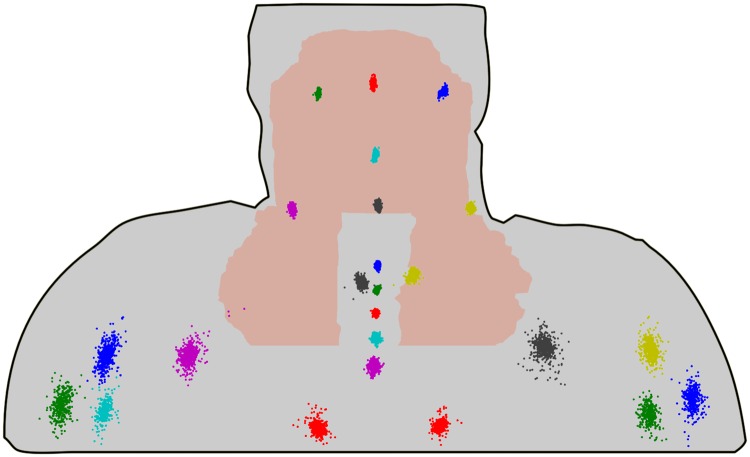
Displacements of the landmarks caused by deformation (all patients, all fractions). For visualization purposes all landmark displacements were projected on the same selected patient representation.

All landmark positions were rigidly transformed with the calculated Procrustes translation and rotation. The residual deformations for each landmark position over all patients are shown in Fig 7. Largest residual motions were found at the shoulders with a mean motion magnitude of (9.2 +-4.7) mm.

### 3.2 Deformation modeling with a biomechanical patient model

Patient specific finite element models with tetrahedrons were created for all patients. The mean number of tetrahedrons needed for meshing the whole patient head neck region was about 60,000.

**Fig 8:**
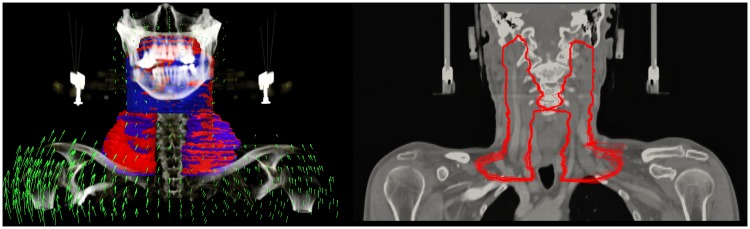
The Calculation of displacement vector fields (DVFs) allows a propagation of the CTV contours from planning image to daily control images. Left: The arrows (green) represent an exemplary DVF of the transformation between a planning CT image and a control CT image. The CTV contour (blue) was propagated to fCTV of the fraction (red). Right: the fCTV of patient #19 propagated to all control CT images are displayed on the planning CT image.

DVFs for all fraction CT images were calculated with the described simulation workflow. Fig 8, left shows an exemplary DVF and the corresponding propagated CTV contours. Fig 8, right shows a coronal cut of the transformed contours though all fractions of this patient. The mean deformation magnitude inside the CTV over all fractions was 1.80 mm over all patients.

### 3.3 Margin generation concepts

Three different margin generation strategies were applied to the CTV to create ITVs for all patients. This took less than 1 s for approach 1 (constant margin), 30 s for approach 2 (variable margin based on distance) and about 90 min for approach 3 (variable margin with biomechanical model).

#### 3.3.1 Size comparison

**Fig 9:**
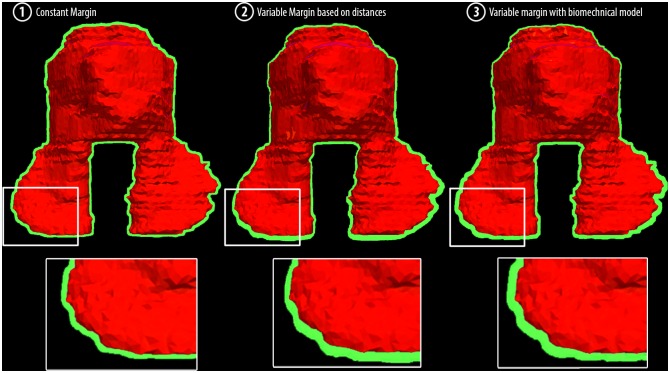
ITV margins (green) of an exemplary patient (#19). Left: Approach 1 with a 3 mm constant margin; center: Approach 2 with a variable margin based on distances; right: Approach 3 with a variable margin approach using biomechanical modeling.

We measured the margin volume (***V***_**ITV \ CTV**_) of the internal margin for each method. The mean volume created with approach 1 was larger than the volume created with approaches 2 and 3 (Table 1). The margin width distribution of the variable margin approaches showed a smaller width at the upper CTV region, where the deformability is low and a larger width for the lower region where the deformability is higher (Fig 9).

**Table 1:** Margin sizes for all three different margin generation methods.

Pat	CTV volume	Internal margin volume		
	*V*_CTV_	*V*_ITV1 \ CTV_ Approach 1	*V*_ITV2 \ CTV_ Approach 2	*V*_ITV3 \ CTV_ Approach 3
1	584.77	159.08	158.45	139.62
2	97.72	36.51	24.67	31.15
3	771.80	202.84	148.30	155.61
4	792.67	189.61	181.58	186.06
5	748.42	152.68	107.35	108.21
6	674.76	171.72	146.23	130.98
7	889.60	193.53	147.39	149.03
8	609.86	140.92	79.12	78.30
9	547.30	125.26	78.62	87.09
10	1191.15	259.89	214.43	200.89
11	1287.35	260.69	240.05	265.84
12	179.22	62.87	51.65	69.43
13	1007.47	207.69	187.81	187.21
14	1121.80	250.62	246.37	273.82
15	519.78	165.80	122.15	129.76
16	1428.01	244.89	235.08	261.61
17	821.66	206.78	160.23	167.45
18	1244.04	219.89	211.25	198.44
19	826.56	225.22	191.68	192.25
**Mean volume**	**807.58**	**182.97 (100%)**	**154.34 (-15.6%)**	**158.57 (-13.3%)**

#### 3.3.2 Healthy tissue exposure and CTV coverage

With the constant margin approach a larger volume of healthy tissue exposed to high doses $\bar{V_{ITV\;\backslash \;fCTV}}$ was measured: Over all fractions and patients (Table 2), a mean ITV volume of 188.6 ml is not located inside the transformed CTV. With the variable margin approaches $\bar{V_{ITV\;\backslash \;fCTV}}$ is reduced to 159.9 and 163.3 ml respectively.

We found a similar CTV coverage for approach 1 and 2 with a mean missed fCTV volume ($\bar{V_{\;\text{fCTV }\backslash \text{ ITV}}}$) of 5.8 and 5.7 ml. Approach 3 missed only 5.0 ml of the fCTV on average.

**Table 2:** Man values and standard deviations of margin metrics for all three different margin generation approaches.

Pat	Volume of healthy tissue inside ITV [ml]			Missed CTV volume [ml]		
	Approach 1 $\bar{V_{\text{ITV}1\;\backslash \text{ fCTV}}}$	Approach 2 $\bar{V_{\text{ITV}2\;\backslash \text{ fCTV}}}$	Approach 3 $\bar{V_{\text{ITV}3\;\backslash \text{ fCTV}}}$	Approach 1 $\bar{V_{\;\text{fCTV }\backslash \text{ ITV}1}}$	Approach 2 $\bar{V_{\;\text{fCTV }\backslash \text{ ITV}2}}$	Approach 3 $\bar{V_{\;\text{fCTV }\backslash \text{ ITV}3}}$
1	158.44 ±1.34	157.51 ±1.41	139.08 ±1.75	1.3 ±1.13	1 ±1.05	1.4 ±1.23
2	36.68 ±0.68	24.98 ±0.99	31.37 ±0.64	0.08 ±0.27	0.21 ±0.62	0.12 ±0.26
3	204.15 ±1.95	150.17 ±2.27	157.31 ±2.43	1.24 ±1.26	1.8 ±1.53	1.63 ±1.24
4	203.79 ±7.42	194.91 ±7.73	194.55 ±5.76	18.02 ±7.73	17.17 ±7.97	12.33 ±6.1
5	152.38 ±1.45	107.57 ±1.8	108.68 ±2.02	0.89 ±1.01	1.4 ±1.37	1.66 ±1.64
6	185.04 ±9.63	161.07 ±11.18	145.21 ±11.28	9.62 ±10.25	11.14 ±11.87	10.53 ±12.01
7	198.32 ±2.93	152.53 ±3.65	154.5 ±3.44	1.19 ±2.21	1.53 ±2.99	1.86 ±2.65
8	138.25 ±1.82	80.82 ±4.4	77.84 ±3.6	2.48 ±2.28	6.85 ±5.06	4.69 ±4.15
9	124.63 ±1.07	78.96 ±1.72	86.57 ±0.9	0.19 ±0.47	1.17 ±1.32	0.31 ±0.37
10	274.02 ±10.83	229.73 ±11.83	215.72 ±13.54	12.27 ±8.44	13.44 ±9.31	12.97 ±9.82
11	265.07 ±7.65	243.66 ±7.41	269.07 ±6.97	3.05 ±4.08	2.27 ±3.41	1.89 ±2.92
12	63.92 ±2.26	53.08 ±2.72	69.82 ±1.13	1.28 ±2.16	1.67 ±2.62	0.63 ±1
13	206.93 ±2.76	185.57 ±2.42	185.1 ±3	3.52 ±3.76	2.05 ±2.54	2.18 ±2.21
14	255.19 ±4.32	249.29 ±3.83	275.12 ±2.96	4.28 ±4.54	2.63 ±3.32	1.01 ±1.25
15	165.89 ±1.43	122.43 ±1.65	130.04 ±1.98	1.21 ±1.08	1.41 ±1.25	1.4 ±1.18
16	254.25 ±7.78	244.07 ±7.72	269.13 ±6.34	4.28 ±4.87	3.91 ±4.93	2.44 ±2.79
17	222.91 ±12.01	175.35 ±12.61	180.95 ±11.05	15.43 ±13.59	14.43 ±14.08	12.8 ±12.39
18	240.19 ±9.87	227.05 ±9.33	215.33 ±8.65	24.12 ±10.29	19.61 ±9.76	20.71 ±8.91
19	232.74 ±5.47	198.38 ±5.23	198.15 ±5.13	5.64 ±4.43	4.82 ±4.09	4.02 ±3.67
**mean**	**188.57 (100%)**	**159.85 (-15.2%)**	**163.34 (13.3%)**	**5.79 (100%)**	**5.71 (-1.3%)**	**4.98 (-14.0%)**

#### 3.3.3 Distance to organs at risk

**Fig 10:**
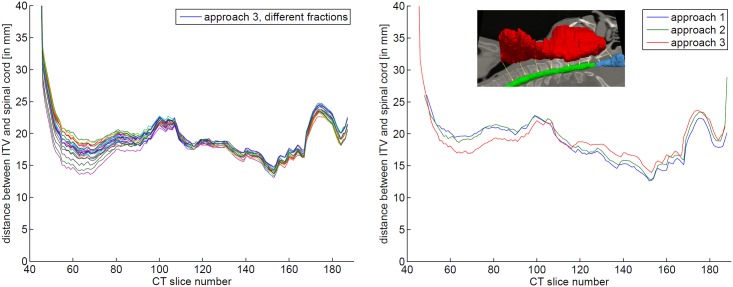
Distance between ITV and spinal cord. Left: distance between ITV and transformed spinal cord ***d***_**SC**_**(*****z*****)** for different fractions of approach 3 for patient #1; right: mean distance between ITV and transformed spinal cord ***d***_**SC**_**(*****z*****)** for different fractions of different approaches for patient #1.

The distance between the ITV and the spinal cord was measured to determine the higher dose exposure risk for this OAR. Since the variable margins (approaches 2 & 3) adapt higher deformability in the lower regions, the margins in those regions are larger and the distance between OAR and ITV is smaller (Table 3 and Fig 10).

**Table 3:** Mean distance between ITV and transformed spinal cord d_SC_(z) of different margin approaches for all patients.

patient	appr. 1	appr. 2	appr. 3
1	12.61	12.66	13.94
2	18.88	19.59	17.27
3	11.76	13.86	13.18
4	10.19	10.81	9.81
5	8.07	10.30	9.17
6	11.88	13.12	12.07
7	10.71	12.71	11.71
8	11.35	13.34	12.53
9	11.60	12.58	12.79
10	7.66	9.66	8.66
11	9.34	10.14	10.86
12	9.71	10.46	10.10
13	9.33	10.70	10.09
14	9.44	10.28	10.55
15	13.58	14.82	14.60
16	11.16	12.70	12.42
17	12.48	12.76	13.47
18	7.13	9.00	8.42
19	12.42	14.37	13.54
mean	**11.02** **100%**	**12.31** **+11.7%**	**11.85** **+7.5%**

## Discussion

We found considerable pose changes for head and neck patients with displacements around 9 mm in magnitude at the shoulder in respect to the skull. Neubauer et al. [46] report similar results for shoulder motion between 2–5 mm in each direction.

The applied method for deformation modeling shows that the pose changes cause considerable motions in the target region (lymph nodes in the lower neck region) with a mean motion magnitude of 1.80 mm.

We can confirm the conclusion of Yang et al. [24]: The distance based variable margins of their approach (approach 2) results in a better target coverage and better OAR sparing than a constant margin (approach 1). Nevertheless, we found only a small increase of target coverage $\bar{V_{\;\text{fCTV }\backslash \text{ ITV}}}$ of 1.3% for our patient cohort. Choosing a higher value than 95% for the confidence interval could improve the coverage but would of course also increase the probability to deposit unnecessary higher doses to the healthy surrounding tissue.

With approach 3 we see a higher increase of 13.3% for target coverage $\bar{V_{\;\text{fCTV }\backslash \text{ ITV}}}$. One reason could be that this approach takes the geometry of each patient into account, whereas approach 2 uses only the distance to landmarks leading to larger margin sizes even if the connecting lines run through air and the landmark displacements have very little effect on the local deformability.

On the other hand: The ITV size is reduced by both variable margin approaches by a comparable amount (15.6% and 13.3%). Applying smaller margins where the expected deformation is relatively small (near the skull) reduces the overall margin size considerably. This can help to spare healthy tissue, especially in the vicinity of the parotid glands and the brain stem [2], and therefore reduce long-term morbidity.

Although the overall size is reduced, the local margin width in regions with high deformability is larger than 3 mm (approach 1), which is necessary to cover the stronger deformation in the lower CTV regions. This might imply a higher risk for OARs. But the risk of higher exposure of the spinal cord as nearest OAR decreased with both variable margin approaches: The mean distance between the transformed spinal cord and the ITV was increased by 11.7% with approach 2 and 7.5% with approach 3. The spinal cord is farther away from the CTV in areas of low deformability and the distance to the spinal cord is large enough to safely increase the margin size to preserve good target coverage. In regions of higher deformability, a reduction of the margin size lowers the risk to the spinal cord while barely worsening target coverage.

We expect only small motions between planning and fraction pose without large changes in joint positions and we ensure consistent poses by avoiding unlikely landmark displacements with the geometric model. Therefore we use homogenous material properties in the biomechanical model for the whole head and neck region. Heterogeneous properties e.g. higher stiffness for the bones could improve the results in the future.

This study was limited on the large deformations due to pose changes. Nevertheless there are additional deformations like soft tissue shifts caused by muscle and organ activities as well as weight loss of the patients, which also need to be addressed in the future.

With our analysis we can confirm the validity of the proposed variable margin construction approach by Yang et al. [24] by an independent modeling approach. The approximation for the automated margin construction in the distance-based approach based on local uncertainties in a patient cohort is fast and applicable in daily clinical routine. Our enhanced method reduces the size of the missed CTV volume markedly (14%) and it shows that the remaining distance between CTV and spinal cord might be overestimated by Yangs et al. [24] approach.

## Conclusion

Compared with the current clinical practice of constant safety margins in radiotherapy, variable margins increase target coverage, reduce risk to OAR and increase healthy tissue sparing at the same time.

Our FE-Model approach independently validates the improvements of the variable margin approach based on landmark distances and increases the target coverage even further.
